# Metabolite profiling and transcript analysis reveal specificities in the response of a berry derived cell culture to abiotic stresses

**DOI:** 10.3389/fpls.2015.00728

**Published:** 2015-09-23

**Authors:** Biruk Ayenew, Asfaw Degu, Neta Manela, Avichai Perl, Michal O. Shamir, Aaron Fait

**Affiliations:** ^1^The Albert Katz International School for Desert Studies, Ben-Gurion University of the NegevBeer-Sheva, Israel; ^2^The French Associates Institute for Agriculture and Biotechnology of Drylands, Jacob Blaustein Institute for Desert Research, Ben-Gurion University of the NegevSede Boqer, Israel; ^3^Department of Ornamental Horticulture, Agricultural Research Organization – Volcani CenterBet-Dagan, Israel; ^4^Department of Fruit Tree Sciences, Agricultural Research Organization – Volcani CenterBet-Dagan, Israel

**Keywords:** abiotic stress, grape, metabolite, transcript, GC–MS, LC–MS, cell culture

## Abstract

As climate changes, there is a need to understand the expected effects on viticulture. In nature, stresses exist in a combined manner, hampering the elucidation of the effect of individual cues on grape berry metabolism. Cell suspension culture originated from pea-size Gamy Red grape berry was used to harness metabolic response to high light (HL; 2500 μmol m^-2^s^-1^), high temperature (HT; 40°C) and their combination in comparison to 25°C and 100 μmol m^-2^s^-1^ under controlled condition. When LC–MS and GC–MS based metabolite profiling was implemented and integrated with targeted RT-qPCR transcript analysis specific responses were observed to the different cues. HL enhanced polyphenol metabolism while HT and its combination with HL induced amino acid and organic acid metabolism with additional effect on polyphenols. The trend of increment in TCA cycle genes like *ATCs, ACo1*, and *IDH* in the combined treatment might support the observed increment in organic acids, GABA shunt, and their derivatives. The apparent phenylalanine reduction with polyphenol increment under HL suggests enhanced fueling of the precursor toward the downstream phenylpropanoid pathway. In the polyphenol metabolism, a differential pattern of expression of flavonoid 3′,5′ hydroxylase and flavonoid 3′ hydroxylase was observed under high light (HL) and combined cues which were accompanied by characteristic metabolite profiles. HT decreased glycosylated cyanidin and peonidin forms while the combined cues increased acetylated and coumarylated peonidin forms. Transcription factors regulating anthocyanin metabolism and their methylation, *MYB, OMT, UFGT*, and *DFR*, were expressed differentially among the treatments, overall in agreement with the metabolite profiles. Taken together these data provide insights into the coordination of central and secondary metabolism in relation to multiple abiotic stresses.

## Introduction

Grape (*Vitis vinifera* L.) is an important crop grown worldwide to produce wine, fresh fruit and derived products (Reisch et al., 2012). Though the crop is important for its economical and health benefits, many abiotic and biotic stresses significantly limit its quality, yield and distribution (Cramer, 2010). One of the topical concerns relating to grapes is the effect of climate change on fruit biochemical attributes defining its quality (Ali et al., 2010). The regulation and modulation of central and specialized metabolism in respect to environmental stresses and vine management has been the focus of significant grape research (Malusa et al., 2004; Dai et al., 2009; Cohen and Kennedy, 2010). That being said environmental cues in the field occur in a combined and complex manner that hampers the understanding of the stress specific regulatory mechanisms involved and the making of appropriate cultural practice for enhanced or reduced effect on fruit traits (Mittler, 2006; Atkinson and Urwin, 2012).

Light and temperature are common environmental cues. Light affects grape berries growth and development though it often occurs with increased temperature under field condition. It is long known that grape berries exposed to sunlight accumulate more anthocyanins, phenolics, and also sugars (Kliewer, 1977). However, exposure derived increased temperature of the berry can lead to degradation of anthocyanin and to a down-regulation of the associated gene transcripts (Haselgrove et al., 2000; Mori et al., 2007) in a development-dependent manner (Mullins et al., 1992; Tarara et al., 2008). Apparently, moderate temperatures and sun exposure enhance anthocyanin accumulation and alter partitioning between anthocyanins and flavonol-glycosides (Downey et al., 2004; Cortell et al., 2007; Matus et al., 2009). Nevertheless, the intimate association between these two environmental cues becomes hardly discernable in the field thus hampering stress specific effects.

Cell cultures provide a very efficient means for studying regulatory cellular processes in plants, to apply and study controlled individual or combined stresses and for producing high-value metabolites (Georgiev et al., 2009; Sharathchandra et al., 2011). The advantages of employing cell cultures are the ease and accuracy in dissecting the different components of complex stresses, in producing of transgenic cell lines, in promoter studies and in monitoring changes in metabolic fluxes (Gollop et al., 2001, 2002; Gaspar et al., 2002; Baxter et al., 2007; Baron and Stasolla, 2008). For instance, Gollop et al. (2001, 2002) showed that Gamay Red cell suspensions derived from fruits, developed and currently maintained in the Perl laboratories, mimic the behavior of genes/promoters/regulatory sequences in grape berries with respect to anthocyanin biosynthesis. Moreover, the system is not restricted to the short maturation season of grape berries and represents a uniform experimental system for exploring the competition between metabolic pathways.

Here we exposed grape berry derived cell cultures from *Vitis vinifera* cv. Gamay Red used in Sinilal et al. (2011) to HL and temperature, separately and combined, measuring in a time series sampling design the changes in their metabolite profile and target gene-transcripts.

## Materials and Methods

The cell suspension culture was established from *V. vinifera* L. cv. Gamay Red berry callus according to D’Onofrio and Boss (2010). The cells were cultivated in Gamborg B5 growth media supplemented with 3% sucrose, 0.1 mg L^-1^ naphthalene acetic acid, 0.2 mg L^-1^ kinetin, 100 mg L^-1^ Myo Inositol and 250 mg L^-1^ Casein Hydrolysate as previously described in Sinilal et al. (2011).

### Experimental Design and Stress Treatments

**FIGURE 1:**
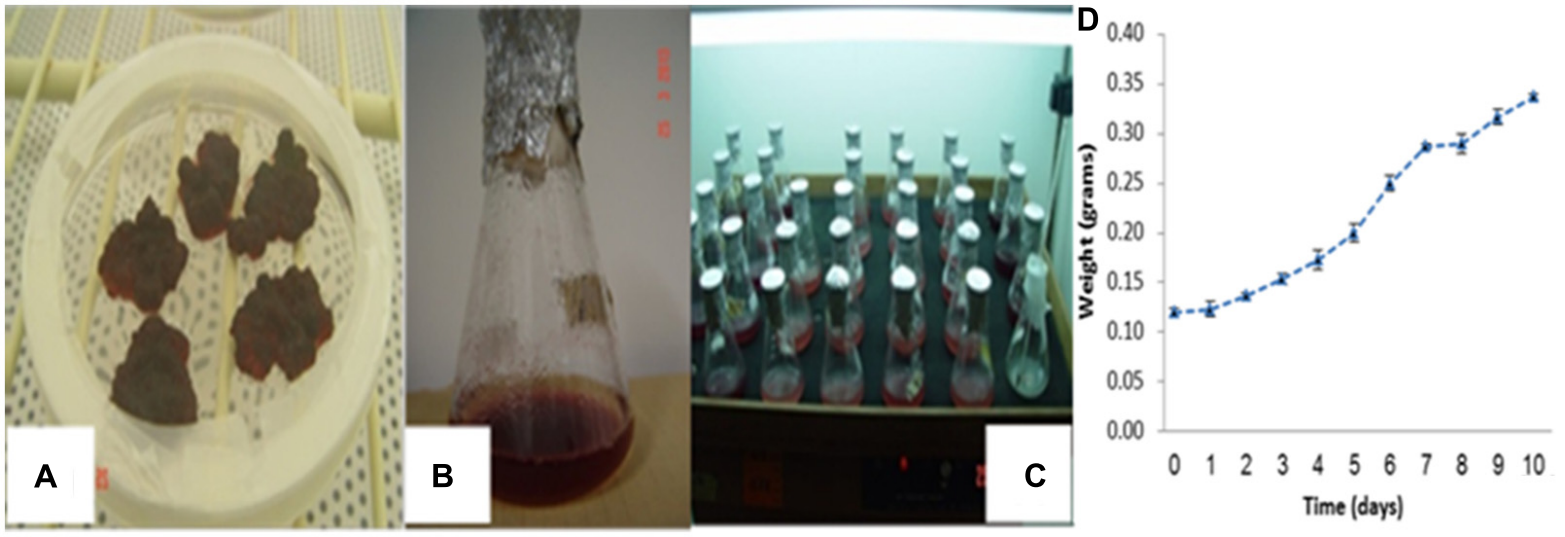
**Dark skin derived callus cultures and its subsequent cell suspension cultures for *in vitro* proliferation. (A)** Initiated callus cultures, **(B)** development of suspension culture, **(C)** cell suspension cultures and their proliferation using 250 mL Erlenmeyer flasks and **(D)** cell growth curve across culture period.

The experimental design is shown in **Figures 1A–C.** Fresh weight growth curve across culture period (**Figure 1D**) and experimental sampling time points was determined prior to laying the experiment. Accordingly, treatments were imposed at the fifth day after culture initiation when the cells enter the rapid growth phase based on fresh weight growth curve observation (**Figure 1D**). Accordingly, sampling time points of 4, 8, 12, and 24 h were selected.

Cell suspension cultures in four replicates were subjected to different treatments. HL intensity, 2500 μmol m^-2^s^-1^, was generated using sequentially ordered 400W Lumen bulbs placed 1 m above the cultures. Temperature was maintained by screening the lamps and by aeration while its measurement was taken using thermometer for monitoring purpose. Furthermore, the lighting devise has its own temperature adjustment system. Control light was set at 100 μmol m^-2^s^-1^ using cool florescent bulbs placed 1m above suspension culture flasks. The light intensity was measured using SS1 SunScan Canopy Analysis System. Using regulated heater, high temperature (HT) was set at 40°C; control temperature was set at 25°C. Combined stress (HLT) was generated by exposing cultures to HL and HT. While filtering samples from growth media using vacuum pump, samples were washed with cold water to avoid carryover chemicals. Cells were immediately frozen in liquid nitrogen and kept at –80°C until metabolite and RNA extraction.

### Metabolite Extraction for Parallel LC–MS and GC–MS Analysis

The samples were extracted for metabolite profiling (LC–MS and GC–MS) as described in Weckwerth et al. (2004). Briefly tissue was grounded using RETCH-mill (Retsch Gmbh, 42787 Haan, Germany) with pre-chilled holders and grinding beads. Forty milligram frozen dried powder were extracted in a pre-chilled methanol/chloroform/water extraction solution (2.5/1/1 v/v/v). Internal standards, i.e., 0.2 mg/mL ribitol in water, 1 mg/mL ampicillin in water and 1 mg/mL corticosterone in methanol were subsequently added. The mixture was then briefly vortexed and ultra-sonicated for 10 min to release the cell components. Subsequently, samples were centrifuged for 10 min at 14000 RPM (micro centrifuge 5417R). The supernatant was mixed with equal volumes (300 μl) of chloroform and Millipore Direct-Q3 UV system purified water, vortexed, and then centrifuged at 14,000 RPM for 5 mins. Finally, 1 mL of water/methanol phase was transferred to UPLC vials for LC–MS and 75 μL were derivatized for GC–MS analysis with micro filtered retention time standard alkane mixture (0.029% v/v *n*-dodecane, *n*-pentadecane, *n*-nonadecane, *n*-docosane, *n*-octacosane, *n*-dotracontane, and *n*-hexatriacontane dissolved in pyridine (0.0075% H_2_O), purchased from Sigma–Aldrich (Jerusalem, Israel) and MSTFA, *N*-methyl-*N*-[trimethylsilyl] trifluoroacetamide purchased from Macherey-Nagel GmbH & Co. KG, Düren, Germany.

#### LC–MS Analysis

For LC–MS analysis, 2 μL of extracted sample was injected to a UPLC–QTOF–MS system equipped with an ESI interface operating in a negative and positive ion mode (Waters Q-TOF Xevo^TM^: Waters MS Technologies, Manchester, UK). Chromatographic separation was carried out on an Acquity UPLC BEH C_18_ column (100 mm × 2.1 mm, 1.7 μm). The auto-sampler and column were maintained at 10 and 40°C, respectively. Up on each run, the mobile phase comprised 95% water, 5% acetonitrile, 0.1% formic acid (phase A), and 0.1% formic acid in acetonitrile (phase B). The solvent gradient program was conditioned according to Degu et al. (2014). Leucine enkephalin was used for lock mass calibration to ensure accuracy and reproducibility, at a concentration of 0.4 ng L^-1^, in 50/50 of acetonitrile/H_2_O with 0.1% v/v formic acid whereby MS conditions were also set according to Hochberg et al. (2013) and Degu et al. (2014). All UPLC solvents were purchased from Bio-Lab Ltd.

#### LC–MS data processing

MassLynxTM software (Waters) version 4.1 was used as the system controlling the UPLC and for data acquisition as described previously (Hochberg et al., 2013; Degu et al., 2014). The raw data acquired were processed using MarkerLynx application manager (Waters) essentially as described previously (Hochberg et al., 2013; Degu et al., 2014). To verify metabolite identification, the database was verified and set using the method described in Degu et al. (2014). Standard libraries described in Arapitsas et al. (2012) were used to validate the annotation of the identified metabolites based on retention time order of commercial standards as essentially explained in Degu et al. (2014). Metabolites were also identified based on a fragmentation pattern searched against the Chemspider metabolite database^1^, standards run in the lab and with previous metabolite annotations (Hochberg et al., 2013, 2015; Degu et al., 2014).

#### GC–MS analysis and data processing

For GC–MS analysis, 1 μL sample was injected onto 30-m VF-5 ms GC column with 0.25 mm i.d., film thickness of 0.25 μm, and +10 m EZ-Guard (Agilent) in splitless mode (32:1). The GC–MS system consisted of an AS 3000 autosampler, a TRACE GC ULTRA gas chromatograph, and a DSQII quadrupole mass spectrometer (Thermo-Fisher ltd). The parameters of the machine were exactly as described in Hochberg et al. (2013). Spectral searching was done by consulting the National Institute of Standards and Technology (NIST, Gaithersburg, MD, USA) algorithm incorporated in the Xcalibur^®^ data software (version 2.0.7) against RI libraries from the Max-Planck Institute for Plant Physiology in Golm, Germany^2^ and finally normalized by respective ribitol amount.

### RNA Extraction and Expression Analysis

Total RNA was extracted using RNeasy Plant Mini Kit (cat. no. 74904) QIAGEN^®^). RNA quality was checked using NanoDrop 2000c UV-Vis Spectrophotometer and formaldehyde treated 1.2% agarose gel electrophoresis. Aliquots were further treated with Baseline-ZERO^TM^ DNase (Cat No. DB0711K) to digest dsDNA and ssDNA before cDNA synthesis. Subsequently, Quanta qScript cDNA was used for cDNA synthesis (Cat No 95047-100).

Primers amplifying 90–100 nucleotides of selected phenylpropanoid pathway and TCA cycle genes were designed for RT-qPCR using Primer Express^®^ primer designing software v2.0 (Applied Biosystems) (**Supplementary Figures S1** and **S2**; Supplementary Table S1). The selected genes and designed primers are listed in Supplementary Table S1 with their details. The designed primers were blasted on NCBI for their specificity and their efficiency was checked using dissociation kinetics performed at the end of each run, using different concentrations standard curve and formaldehyde treated 1.2% agarose gel electrophoresis (**Supplementary Figure S2**).

RT-qPCR was done using Applied Biosystems StepOnePlus^TM^ Real-Time PCR using PerfeCTa^®^ SYBR^®^ Green Fast Mix^®^ (Quanta Biosciences), 50 ng cDNA and 300 nM of gene-specific primer in a final volume of 10 μL. PCR amplification was performed using the following conditions: 40 cycles of denaturation at 95°C for 15 s, annealing at 60°C for 15 s and extension with 72°C for 20 s. All reactions were performed in technical triplicates and relative quantification values for each target gene were calculated by the 2^-ΔΔCT^ method (Livak and Schmittgen, 2001). Ubiquitin was used as an internal reference gene for comparing data from different PCR runs or cDNA samples (Dekkers et al., 2012).

### Statistical Analysis

LC–MS and GC–MS data were normalized (to internal standards and tissue weight) and subjected to Bonferroni correction before comparing. Comparisons between treatments at each sampling time and over all experimental period were performed using the Student’s *t*-test. All the statistical analyses were calculated using the R-software environment R 3.0.1^3^.

## Results

### GC–MS Based Central Metabolism

Central metabolites change in response to HL, HT, and their HLT during the four exposure times were normalized to their respective control and are expressed in (fold changes).

**FIGURE 2:**
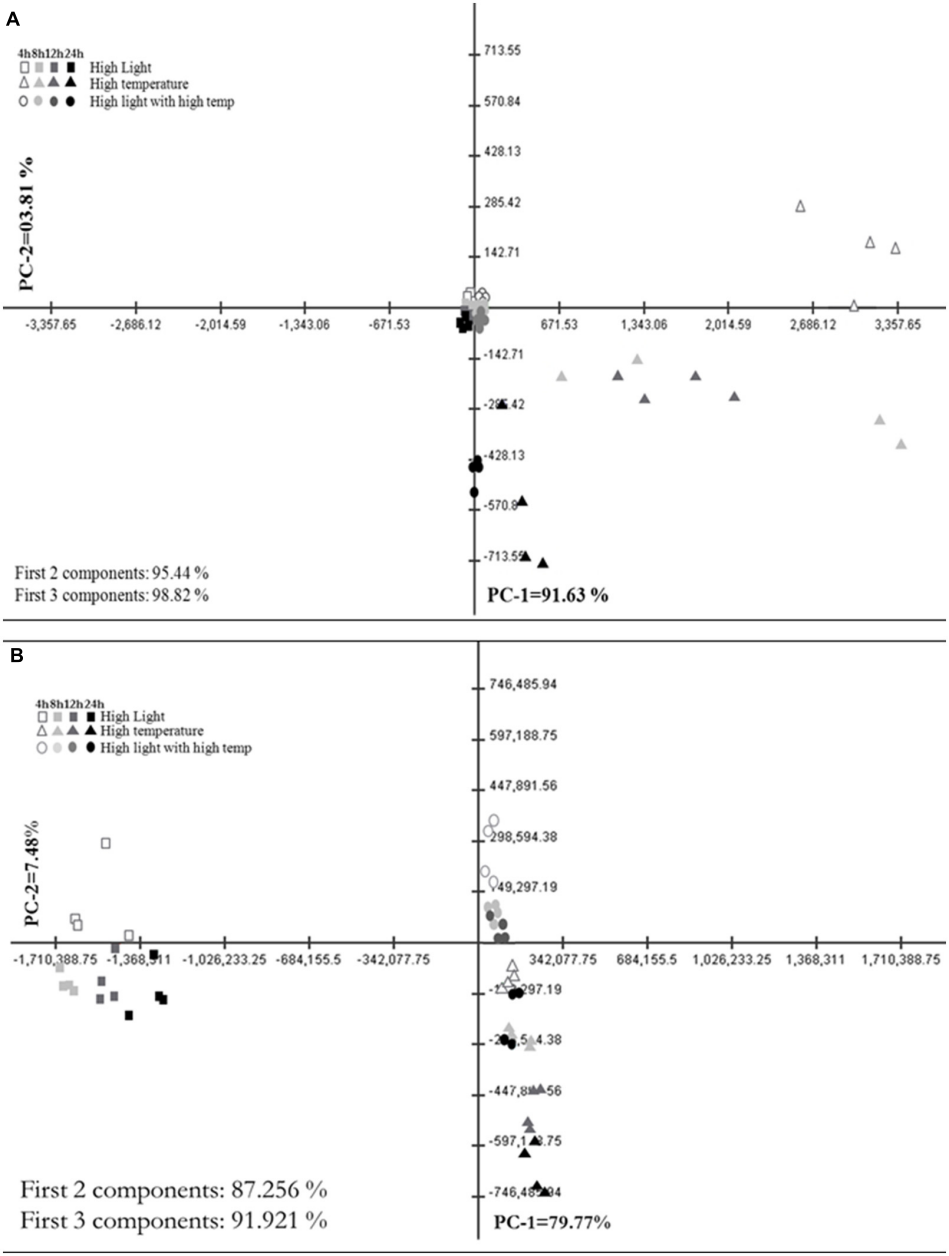
**Metabolic shifts in specialized metabolites under high light (HL), high temperature (HT), and combined stress (HLT) across time**. Principal Component Analysis (PCA) plot of PC-1 vs. PC-2 of metabolite profile obtained from **(A)** GC–MS and **(B)** LC–MS–QTOF stressed grape suspension cultures after 4, 8, 12, and 24 h of exposure. Percentage of the variance by each principal component is indicated on respective axis. Each points represent biological samples (*n* = 4)

Principal Component Analysis (PCA) plot of GC–MS based annotated metabolite profile showed the difference among HL, HT, and HLT treatments over time (**Figure 2A**). Accordingly, HT and HLT treated samples exhibited clear separation across time while HL treated samples clustered together (**Figure 2A**). The first two and three principal components accounted for 95.4 and 98.8%, respectively of the total variance and discriminated treatments and time of exposure. The first principal component (PC1) contributed 91.6% of the variance and clearly separated HT from HLT and HL. HL and HLT grouped closer to each other than to HT. The separation along PC1 was mostly due to changes in succinate, alanine, glycerate, serine, and shikimate (Supplementary Table S2). PC2 and PC3 accounted for a significantly lower portion of the variance, 3.8 and 3.4%, respectively, separating HT samples over time and at the fourth time point HLT from the other samples (Supplementary Table S2).

**FIGURE 3:**
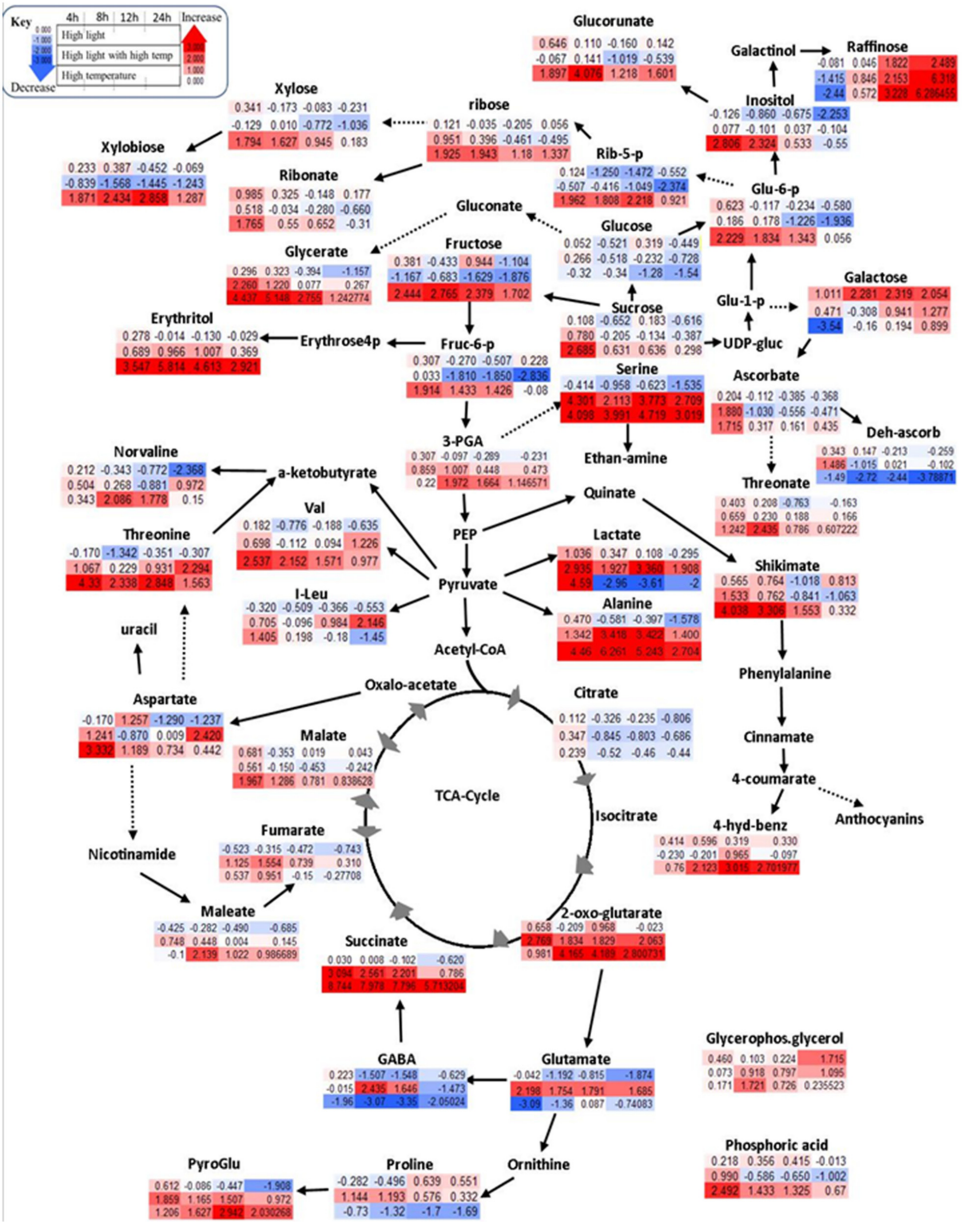
**Schematic representations of primary metabolites in fold change from respective time point control in Log2 values**. Different colors represent levels of metabolite fold change where red is increasing and blue is decreasing. Mean values are presented (*n* = 4). Each row (upper row = HL treatment, middle row = combined HL with high temperature, lower row = HT) shows fold change from respective time point control while each column is different time points after treatment application (4, 8, 12, and 24 h).

A communal and rapid decrease in sugar reserves, pentose phosphate pathway (PPP) and glycolysis intermediates were observed during HL and combined stresses (**Figure 3**; Supplementary Table S3). Apart from these, HL stimulated the accumulation of stress related sugars such as raffinose and galactose and a significant reduction in amino acid content during the experimental period (**Figure 3**; Supplementary Table S3). TCA cycle intermediates such as maleate, aspartate, and TCA cycle associated GABA shunt were not affected significantly under HL stress showing a general trend of decreasing relative content over time (**Figure 3**; Supplementary Table S3).

High temperature lead to increased sugars and phosphates associated with the non-oxidative phase of PPP (**Figure 3**; Supplementary Table S3), e.g., increased fructose 6-phosphate, glucose 6-phosphate, 3-phosphoglycerate, and ribose 5-phosphate significantly unlike HL or combined stress. Longer exposure to HT caused a gradual reduction of the above mentioned metabolites with a marked increase of xylose and xylobiose, prevalently derived from ribose. Other PPP derivatives, e.g., erythritol, glycerate, ribose, and ribonate, were also significantly increased under HT (**Figure 3**; Supplementary Table S3). Amino acids alanine, serine, valine, and threonine accumulated in response to HT while glutamate, GABA, proline, and isoleucine were decreased (**Figure 3**; Supplementary Table S3). The change in TCA cycle intermediates under HT stress included the accumulation in 2 oxoglutarate, succinate, and malate, while citrate was reduced (**Figure 3**; Supplementary Table S3).

HLT stress showed distinct responses unlike its components of HL and HT stresses. Specifically, cell membrane structural components such as xylose and its oligomer, xylobiose, decreased significantly under combined stress differently, while sugar derivatives, glucorunate, and raffinose, accumulated (**Figure 3**; Supplementary Table S3). Accumulation of amino acids such as alanine, valine, serine, proline, isoleucine, phenylalanine, aspartate, and threonine was observed over time (Supplementary Table S3). Unlike HL and HT, combined stress also significantly increased glutamate and its derived γ-aminobutyrate, GABA, proline, and pyro-glutamate. In the TCA cycle, the combined stress significantly decreased malate and citrate while increasing 2-oxoglutarate, succinate, and fumarate (**Figure 3**; Supplementary Table S3). Organic acids such as maleate were also increased in under these conditions.

### LC–MS Based Polyphenol Metabolism

Principal Component Analysis plot of LC–MS based metabolite profiling that comprised of more than 7000 chemical features, depicts a distinct difference of HL, HT, and HLT treatments over time (**Figure 2B**). The first two and three principal components accounted for 87.3 and 91.9%, respectively of the total variance and discriminated treatments and time of exposure. The PC1 contributed 79.8% of the variance and clearly separated the samples mainly on the basis of the treatment. Phenylalanine, cyanidine 3-*O*-glucoside and peonidin 3-*O*-(6-p-coumaroyl)-glucoside had the highest contribution to the sample separation (Supplementary Table S2). PC2 accounted for 7.5% of the variance within the dataset, separating samples on the basis of the duration of the treatment and sorting out the HT treatment from combined stresses (**Figure 2B**). Glycosylated, acetylated, and coumaroylated forms of peonidin and cyanidin had the highest eigen values explaining major differences between treatments and time samples along PC2 (Supplementary Table S2). PC3 was comparatively negligible (4.7% of the variance).

**FIGURE 4:**
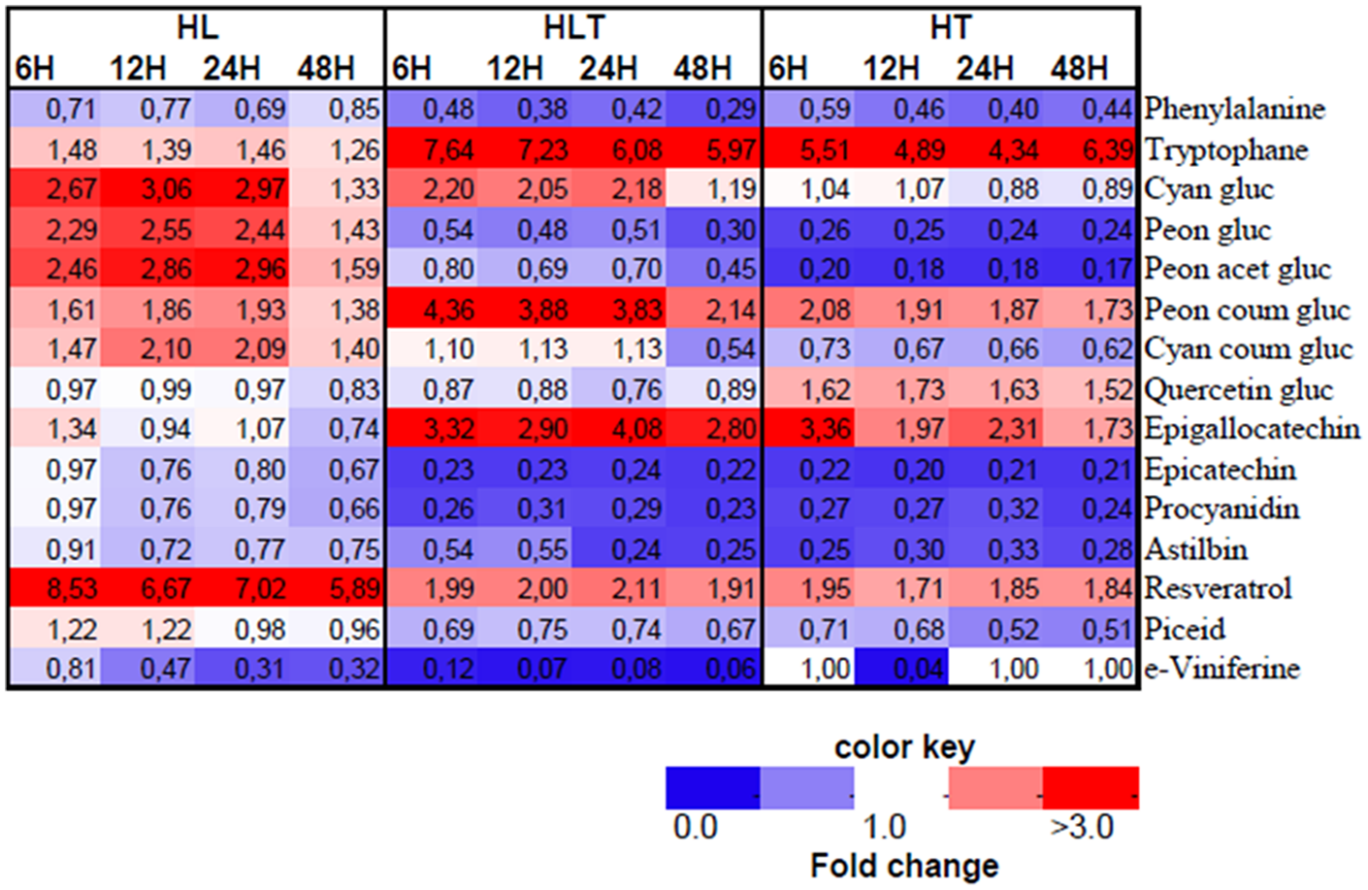
**Heatmap of annotated LC–MS based metabolites in fold change from respective time point control**. Treatments exposed to HL = 2500 μmol m^-2^s^-1^ light intensity, HT = 40°C, HLT = HL with HT for 4, 8, 12, and 48 h. Different colors represent the increase (red) or decrease (blue) of the metabolites fold change as indicated in the color key. Mean values are presented (*n* = 4).

The annotated LC–MS profile revealed a significant change in the phenylpropanoid pathway in response to HL, HT, and HLT (**Figure 4**). There was a communal phenylalanine decrease in all the treatments over time though they showed differential branching in the downstream metabolites distinct to particular stress.

Upon exposure to HL, in the phenylpropanoid pathway, accumulation of cyanidin-type of anthocyanins was observed over time, e.g., acetylated and coumarylated cyanidin and peonidin forms (**Figure 4**). Concurrently, HL caused a five- to eightfold accumulation from control in resveratrol, and its derivative piceid, which are synthesized at the early branching pathways of phenylpropanoid. The fold change from control in astilbin, one of major dihydro-flavonol glucosides in grape was found significantly higher in HL treated cell cultures followed by combined stress.

High temperature caused a significant induction (four- to sixfold change) of tryptophan accumulation. In addition, under HT epigallocatechin and quercetin-3-*O*-glucoside increased during the first hours of exposure, while declining progressively over time (**Figure 4**). In contrast, a decrease of cyanidin-3-*O*-glucoside abundance was measured throughout the experiment. Also peonidin derivatives of anthocyanin in glucosylated and acetylated forms were significantly reduced over time while *p*-coumaroylated peonidin was increased. Furthermore, coumaroylated cyanidin was reduced steadily in a time-dependent manner as exposed to HT, but not to HL or combined stress (**Figure 4**). In addition, astilbin and procyanidin were significantly reduced right after application of the treatment (within the first 4 h) and stayed significantly low over time (**Figure 4**).

Combined stress resulted in distinct metabolic profile from its component stresses (**Figure 3** and **4**). An additive five- to sevenfold increment of tryptophan relative content was observed over time in combined stress (which in relative magnitude was followed by HT, four- to sixfold and HL showing only a mild change 1.4 to 1.5-fold) (**Figure 4**). Combined stress led to a selective increment of dihydroxyaled anthocyanins concentration whose major components are glycosylated and coumarylated cyanidin. This effect was likely due to the light component, which showed similar results when taken individually (**Figure 3**). Combined cue also resulted in a significant higher accumulation of coumarylated peonidin higher in magnitude when compared to the effect of the stresses applied individually (**Figure 4**). On the other hand, the decrease in content of glucosylated and acetylated forms of peonidin were likely driven by the temperature component which had a similar effect, while HL stress induced their accumulation (see above). A similar “temperature-driven” effect of the combined stress was measured for piceid and resveratrol which accumulated in respect to the control (**Figure 4**).

### RT-qPCR Based Transcript Analysis

**FIGURE 5:**
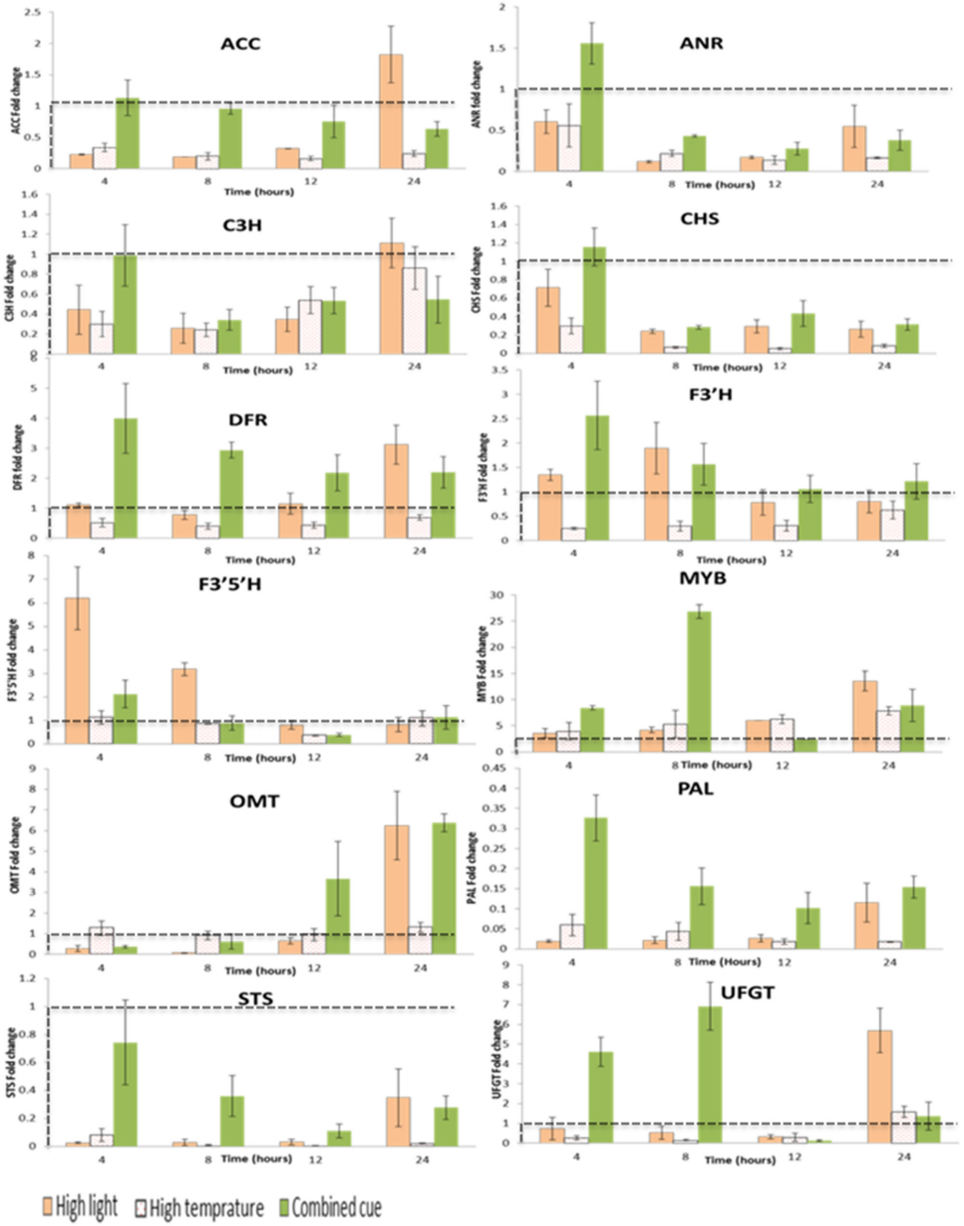
**Fold change transcript levels of enzymes and transcription factors mediating phenylpropanoid pathway from respective time point control**. Fold change values between zero and one (below the dashed line) indicates lower transcrip level compared to the control. *ACC*, Acetyl-CoA carboxylase 1-like; *ANR*, Anthocyanidin reductase; *C3H*, Coumarate 3-hydroxylase; *CHS*, Chalcone synthase; *DFR*, Dihydroflavonol 4-reductase; *F3′H:*flavonoid 3*′* hydroxylase; F3*′*5*′*H: flavonoid 3*′*,5*′* hydroxylase; *MYB*, Myb-related transcription factor; *OMT, O*-methyltransferase; *PAL*, Phenylalanine ammonia lyase; *STS*, Stilbene synthase; *UFGT*, UDP flavonoid 3-*O*-glucosyltransferase; *n* = 3, mean values ± SE.

Transcripts responsible for mediating phenylpropanoid pathway were expressed differently between treatments (**Figure 5**; Supplementary Table S4) and associations between the level of expression of specific genes and related metabolites were observed, particularly in relatively downstream biochemical reactions.

The enzyme involved at the beginning of the phenylpropanoid pathway, phenylalanine ammonia-lyase (*PAL)*, was down-regulated in all treatments despite a general increased abundance of downstream metabolites (**Figure 5**). Similarly, in spite of the increased accumulation in stilbenoid and proanthocyanidins, stilbene synthase (*STS)*, and anthocyanidin reductase (*ANR*) were down-regulated.

That being said, in accordance to the overall accumulation of anthocyanins, the first enzyme committed to anthocyanin biosynthesis in the phenylpropanoid pathway, dihydroflavonol-4-reductase *(DFR)*, was increased under HL condition. Interestingly a significantly increased transcript level of *F3′5′H* and *F3′H* was observed at the beginning of the treatments and decreased at the later stages in HL condition (Supplementary Table S4). The fold change magnitude of *F3′5′H* is significantly higher than *F3′H* (up to sixfold) in contrast to the respective proportions of tri- and di-hydroxylated anthocyanins. The concomitant up-regulation in *DFR* and *F3′H* supports the higher accumulation of cyanidin-type anthocyanins under HL condition. Also transcription factors related to polyphenol metabolism were up-regulated under HL stress, among them, MybA1 (Myb-related transcription factor), *O*-methyltransferase, *OMT*, and UDP flavonoid 3-*O*-glucosyltransferase, *UFGT* (**Figure 5**; Supplementary Table S4).

Upon exposure to HT, a general significant down-regulation of pehenylpropanoid pathway mediating transcripts was observed supporting the reduced metabolites levels in this pathway (**Figure 5**; Supplementary Table S4). In line with this, we observed a significant up-regulation of MybA1 and this was accompanied by increased in coumarylated peonidin amount. This is also associated with a slight increment of *OMT* transcript levels in a steady manner over period of time not seen under highlight or combined stress.

The transcript levels of genes mediating phenylpropanoid pathway metabolites during combined stress showed significant differences in expression level from its component stresses. A significant time-dependent change in expression of Acetyl-CoA carboxylase 1-like, *ACC*, was observed, whereby an initial up-regulation was followed by a gradual down regulation over time. This is in contrast to HL stress inducing an initial down-regulation followed by a gradual increment in transcript level. Unlike HL, combined stress enhanced *F3′H* transcription more than *F3′5′H* during the period of the treatment. This change in transcript level is in line with the enhanced annotated anothocyanins. The consistently significant up-regulation of *DFR* (two- to fourfold increases) further confirms the enhancement of this pathway. Furthermore, transcription factors related to polyphenol metabolism such as MybA1*, OMT*, and *UFGT* were also significantly up-regulated in a similar manner in response to HL stress (**Figure 5**; Supplementary Table S4).

**FIGURE 6:**
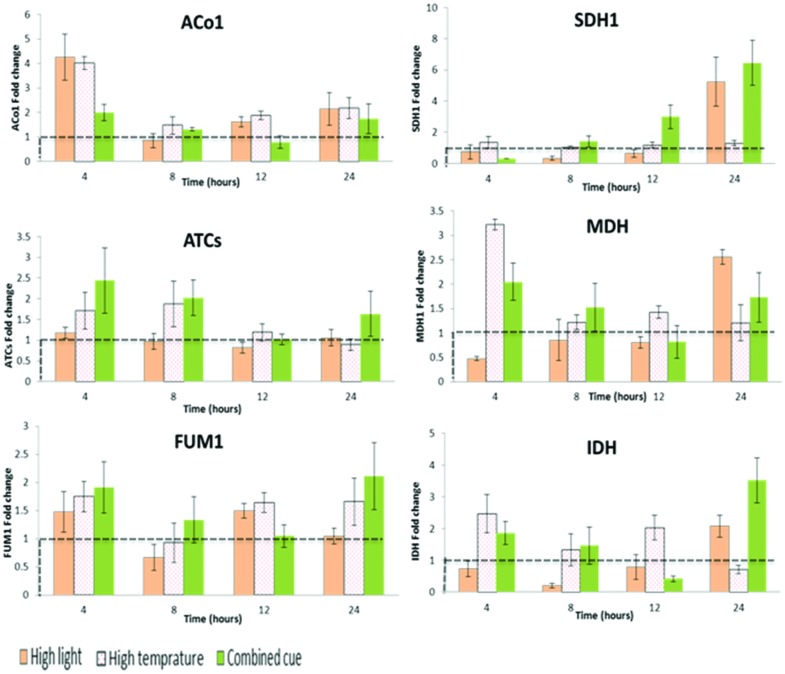
**Fold change in transcript levels of TCA cycle mediating enzymes from respective time point control**. Fold change values between zero and one (below the dashed line) indicates lower transcrip level compared to the control. *ACo1*, Aconitate hydratase 1-like; *SDH1*, Succinate dehydrogenase; *ATCs*, Citrate synthase; *MDH*, Malate dehydrogenase; *FUM1*, Fumarate hydratase; *IDH*, Isocitrate dehydrogenase; *n* = 3, mean values ±SE.

TCA cycle transcripts enhanced though their expression level was not significant between the treatments (Supplementary Table S4). Apparently, *ATCs* and *ACo1* and *IDH* display a trend of increase in their expression level during combined and HT stresses (**Figure 6**).

## Discussion

The impact of various environmental cues on grape berries metabolism is diverse and is further complicated by the cultivar differences and by the occurrence of developmental stage specific responses (Downey et al., 2003, 2004; Cramer et al., 2011; Flamini et al., 2013). Understanding how berries respond to multiple environmental cues is of paramount importance to adopt appropriate cultural practices to obtain a desired effect on the quality of the final product, the wine. We investigated the regulation of isolated HT, HL, and HLT effect on dark colored grape suspension cultures using metabolite profiling and targeted transcript analysis. The model system, cell suspension culture, revealed a differential response in berry metabolism and transcript level (**Figure 3**), whereby the biochemistry of the berry was driven preferentially by either one of the component stresses.

### High Light Enhances Polyphenol Metabolism

The focus of many researches in grape has been polyphenol metabolism due to its significance in nutrition and in determining wine quality (Cohen and Kennedy, 2010; He and Giusti, 2010). Polyphenol metabolism is responsive to abiotic stresses likely as a protective and defense strategy (Cohen et al., 2012; Teixeira et al., 2013; AbdElgawad et al., 2014).

In this study, isolated and combined treatment showed differential regulation of polyphenol metabolism and their associated transcript level. HL caused the accumulated of stilbenoids, proanthocyanidin, and dihydroxylated (cyanidin-form) anthocyanin. Unlike HL and HLT, increased temperature led to a decrease in anthocyanins, down-regulating their biosynthesis. This finding is in agreement with the results reported by Mori et al. (2007), showing degradation of anthocyanin in response to HT using softened grape berries.

Interestingly, all treatments enhanced biosynthesis of dihydroxylated anthocyanins perhaps at the expenses of trihydroxylated anthocyanins. This observation was common to the ‘control’ cultures during the experimental period, and the transcript levels of *F3′H* and *F3′5′H* were in agreement with their respective metabolite profile. Competition between di and trihydroxylated anthocyanins forms was observed following UV-B exposure of berries (Martinez-Luscher et al., 2014). In addition, a similar interplay was shown by Bucchetti and Intrieri (2007) in several red varieties, whereby specifically three-substituted anthocyanins decreased in response to low light conditions. Hence the relation between the two branches of flavonoid metabolism seems to be highly inter-regulated and responsive to general environmental cues. On the other hand, reports from Castellarin and Di Gaspero (2007) showed that the enhancement and accumulation of tri- and dihydroxylated anthocyanins is associated with varietal differences at transcript level. In their report, cultivars bearing dark berries with violet and blue hue were associated with tri-hydroxylated anthocyanins as compared to red-skinned cultivars. Furthermore, anthocyanin profile of 50 red table grape cultivars by Pomar et al. (2005) showed an increment of dihydoxylated anthocyanins in abundance than trihydroxylated while Mattivi et al. (2006) demonstrated the significant correlation between these partitioning in red and white varieties leading to difference in anthocyanin composition and pattern of other metabolites such as flavonols and flavanols. To summarize, while the genetic component has a major influence in anthocyanin partitioning, these studies suggest that environmental cues can dictate the metabolic phenotype.

### Reduction of Sugar Reserves in Isolated and Combined Cues

Concentration of sugar reserves and their intermediate monosaccharides were generally reduced in response to HL, HT, and HLT. Under such conditions, sugar reserves can sustain anthocyanin biosynthesis as well as stress related responses such as ABA accumulation regulated through berries temperature (Mori et al., 2005; Deluc et al., 2009). In addition, associated oligosaccharides including raffinose were increased. Previous studies showed a similar increment of raffinose family oligosaccharides in response to oxidative stress (Nishizawa et al., 2008a; Pillet et al., 2012; Elsayed et al., 2014). It is suggested that raffinose and associated oligosaccharides act as cellular protectants during stress due to their scavenging activity of hydroxyl radicals and liposome protection (Busch et al., 2005; Nishizawa et al., 2008b).

Combined light and heat, is a common phenomenon in the field. Previous studies in a vineyard also found accumulation of raffinose during berry maturation (Degu et al., 2014). This is consistent with the induction of RFO metabolism during water loss events as shown in different species and organs (Taji et al., 2002; Peters et al., 2007). Sugars respiration using pathways of glycolysis, PPP, and TCA cycle provides energy and reducing power (Dai et al., 2013) as well as precursors for the synthesis of organic acids, amino acids, anthocyanins and other secondary metabolites (Centeno et al., 2011; Dai et al., 2013).

As berries are exposed to different cues, one protection mechanisms is cell wall lignification (Toivonen and Brummell, 2007). Accordingly, the observation of increased cell wall components xylose and xylobiose, in response to HT suggests the induction of a protection mechanism. In addition, evidence for heat-stress specific increased of PPP intermediates is in support of previous reports in *Arabidopsis* showing induction of PPP-related transcripts in response to heat stress (Rizhsky et al., 2004). The activation of this pathway has been associated with strategies of reducing oxidative stress and regulating redox homeostasis (Stincone et al., 2014).

### High Temperature and Combined Stress Induced Amino Acids

Among the different strategies used in cells to cope with stress, accumulation of amino acids is common (Lugan et al., 2010; Krasensky and Jonak, 2012). Grape leaves exposed to terminal drought treatments accumulated amino acids, in particular proline (Hochberg et al., 2013) and grape berries showed a similar accumulation (Hochberg et al., 2015). Kaplan et al. (2004) and Sweetman et al. (2014) suggest that the accumulation of amino acids during increased temperature in fruits is to sustain acclimation and provide energy reserves. In line with this hypothesis, our results show that the increase in the level of amino acids was associated with that of TCA cycle intermediates and contribute to the cellular energy balance. Sweetman et al. (2014) further investigated using proteomic analysis confirmed that the increment of amino acids during heat stress in grapes was not associated with protein degradation rather *de novo* amino acid biosynthesis. Intriguingly, our results show a contrasting effect of temperature and light on amino acids.

The induction of Glu-derived metabolism GABA and Proline, two osmo-protectants, is likely related to stress (Sweetman et al., 2014). GABA is suggested to have a role during abiotic and biotic stress response (Kinnersley and Turano, 2000; Sweetman et al., 2014) and also on carbon and nitrogen metabolism (Fait et al., 2007). During abiotic stress, the shunt is proposed to have a role by-passing a down-regulated TCA cycle (Fait et al., 2007; Sweetman et al., 2014). Interestingly enough, the combined cue and HT stresses increased the content of GABA shunt related metabolites, but not so for HL intensity. Our results suggest that temperature induces the shunt and support the conclusions by Sweetman et al. (2014) that the heat component in a vineyard exposed to increased sun radiation leads to the changes observed in the amino acids.

### TCA Cycle Partial Enhancement and Organic Acid Metabolism

There was interplay among sugars, amino acids, TCA cycle metabolites, and organic acids in response to different environmental cues (Araujo et al., 2012). In our study, the decarboxylation part of TCA cycle displayed a reduction in intermediate content in all the treatments while HL led to a decrease in content of intermediates of the second part of the TCA cycle. Apparently, it was found that malate and citrate concentration was degraded during combined cue while isolated HL affected less. This is in agreement with Spayd et al. (2002) that investigated the impact of HT on exposed grape bunches at different developmental stages. They suggested reduction in malate is associated with increased respiration with HT. Similarly, malate reduction in grape berries was reported in response to increased night time temperature due to increased respiration (Sweetman et al., 2009, 2014). Here, the reduction in decarboxylation part of TCA cycle was associated with increased 2-oxoglutarate and associated γ-aminobutyrate, GABA shunt.

Kaplan et al. (2004) showed the increased accumulation of succinate in HT stressed *Arabidopsis* plants. Similarly, we have found an increased level of succinate leading to subsequent fumarate with HT containing treatments. Succinate is involved with mitochondrial electron transport via four inner-membrane protein complexes towards oxygen with concomitant translocation of protons in to the inter-membrane space creating a potential across the inner membrane to be used for ATP synthesis (Fernie et al., 2004).

### Transcriptional Regulation of Metabolite Response to Combined and Isolated Environmental Cues

Phenylpropanoid pathway transcripts were expressed differently among treatments although we could not find a general strong association between the level of expression of metabolic genes and related metabolites. Not surprisingly in previous studies the presence of non-transcriptional regulatory mechanism in the phenylpropanoid pathway has been indicated (Sreelakshmi and Sharma, 2008). For example increased level of downstream metabolites in the pathway was not associated with *PAL* transcript level. This is in line with our study showing reduced transcript levels of *PAL* (**Figure 5**; Supplementary Table S4) but enhanced downstream phenylpropanoid metabolism.

Under HL, the enhanced accumulation of stilbenoids and proanthocyanidin is not in agreement with their transcript levels, i.e., reduced level of *STS* and *ANR*. This could be associated with the enzymes kinetics and/or post transcriptional regulation. On the other hand the accumulation of cyanidin-form of anthocyanin corresponds with increased *F3′H* and *DFR* transcript level, a committed step towards anthocyanin biosynthesis. The coordinated regulation of anthocyanin metabolism in grapes was reported by Ramazzotti et al. (2008). Furthermore, isolated HL stress induced downstream metabolite biosynthesis associated with increased *OMT, MYB*, a *UFGT* positive transcription regulator (Ravaglia et al., 2013), and *UFGT* transcript levels. Transcript (Castellarin and Di Gaspero, 2007) and protein (Lücker et al., 2010) level studies indicated a high correlation of OMT with the expression of other flavonoid pathway genes and proteins at onset of ripening in several *V. vinifera* cultivars. In addition, Castellarin and Di Gaspero (2007) showed OMT is involved with 3′ and 5′ position methylation of anthocyanidin-glucosides on the B-ring of the flavan skeleton. Furthermore, the authors showed an induced expression of MYB and UFGT which is highly correlated with grapes at maturity stage for anthocyanin biosynthesis (Castellarin et al., 2007).

Early induction in *MYB*, *UFGT*, and *OMT* was also shown under combined stress, supporting increased in acetylated and coumaroylated anthocyanin forms. Nevertheless, the decreased relative content of trihydroxylated anthocyanin (delphinidin-form) in contrast to dihydroxylated anthocyanin abundance did not match the higher *F3′5′H* transcription level, suggesting a post-transcriptional regulation of this branching point.

Overall the transcript analysis together with the metabolite data suggest a strong input of C resource to sustain the substantial accumulation of dihydroxylated anthocyanins under HL condition followed by combined stress, while HT down-regulated their biosynthesis with a further degradation of the available anthocyanins.

## Conclusion

Dissecting environmental cues to their components is fundamental to understand the driving force and underlying regulatory mechanisms of the effect on grape berry metabolic profile. The integration of metabolite changes and transcript level reveals stress-associated metabolism. In general, the present finding is in agreement with the field reports that suggest an overall decrease in sugar reserve (Mori et al., 2005; Deluc et al., 2009), and an increase in cell wall components (Toivonen and Brummell, 2007), amino acids (Lugan et al., 2010; Krasensky and Jonak, 2012), and partial TCA metabolites (Kaplan et al., 2004) under stress. While increased in polyphenol characterized the response to HL, HT, and HLT reduced most anthocyanins and down regulated their biosynthesis. However, the increase in malate and fraction of antocyanins in response to individual and combined stress contrast that of the field reports (Mori et al., 2007; Sweetman et al., 2014). These results could be due to the short-term treatments used in the present study, or to the fact that, in the field, the effect of surface temperature and light are not easily discernable. Thereof studying stress responses using berry derived cell cultures can significantly contribute to testing isolated environmental components and other cues, e.g., elicitors and hormones, and model the behavior of berries in the field.

## Conflict of Interest Statement

The authors declare that the research was conducted in the absence of any commercial or financial relationships that could be construed as a potential conflict of interest.

## Footnotes

1 http://www.chemspider.com/

2 http://www.mpimp-golm.mpg.de/mms-library/

3 http://www.r-project.org/.

## Supplementary Material

FIGURE S1 **| Selected mediating enzymes and transcription factors in phenylpropanoid pathway and TCA cycle used in RT-qPCR based transcript analysis adapted from Zabala et al. (2006).** *ACC*, Acetyl-CoA carboxylase 1-like; *ANR*, Anthocyanidin reductase; *C3H*, Coumarate 3-hydroxylase; *CHS*, Chalcone synthase; *DFR*, Dihydroflavonol 4-reductase; *F3′H*, flavonoid 3′ hydroxylase; F3′5′H, flavonoid 3′,5′ hydroxylase; *MYB*, Myb-related transcription factor; *OMT, O*-methyltransferase; *PAL*, Phenylalanine ammonia lyase; *STS*, Stilbene synthase; *UFGT*, UDP glucose-flavonoid 3-*O*-glucosyl transferase; *ACo1*, Aconitate hydratase 1-like; *SDH1*, Succinate dehydrogenase; *ATCs*, Citrate synthase; *MDH*, Malate dehydrogenase; *FUM1*, Fumarate hydratase; *IDH*, Isocitrate dehydrogenase; *n* = 3, mean values ± SE.

FIGURE S2 **| Specificity of the designed primers for transcript analysis and their negative control, formaldehyde treated 1.2% agarose gel electrophoresis, each lane represents specific primers and ntc, lane 1 and 20 are size markers in all the three gels.** First gel electrophoresis: *ANR, CHS, ATCs, UBQ10-1, UBQ10-2, IDH, MDH, PAL, SDH, STS, UFGT, ACC, C3H, F3H, DFR, FUM* from 2 till 19 sequentially, Second electrophoresis: *MYB, ACT, F3′5H, ACo1-ntc, ANR-ntc, CHS-ntc, UBQ-10-ntc, IDH-ntc, MDH-ntc, OMT-ntc, PAL-ntc, SDH-ntc, STS-ntc, UFGT-ntc, ACC-ntc, C3H-ntc, F3H-ntc, DFR-ntc* from 2 till 19 sequentially, third electrophoresis: *FUM-ntc, MYB-ntc, ACT-ntc, F3′5′H-ntc, UBQ-10-1-ntc* from 2 till 6 respectively.
