# Supplementary figures and images for: Metabolite profiling and transcript analysis reveal specificities in the response of a berry derived cell culture to abiotic stresses
Source: Front Plant Sci. 2015 Sep 23;6:728. doi: 10.3389/fpls.2015.00728 (PMC4585150; doi:10.3389/fpls.2015.00728)

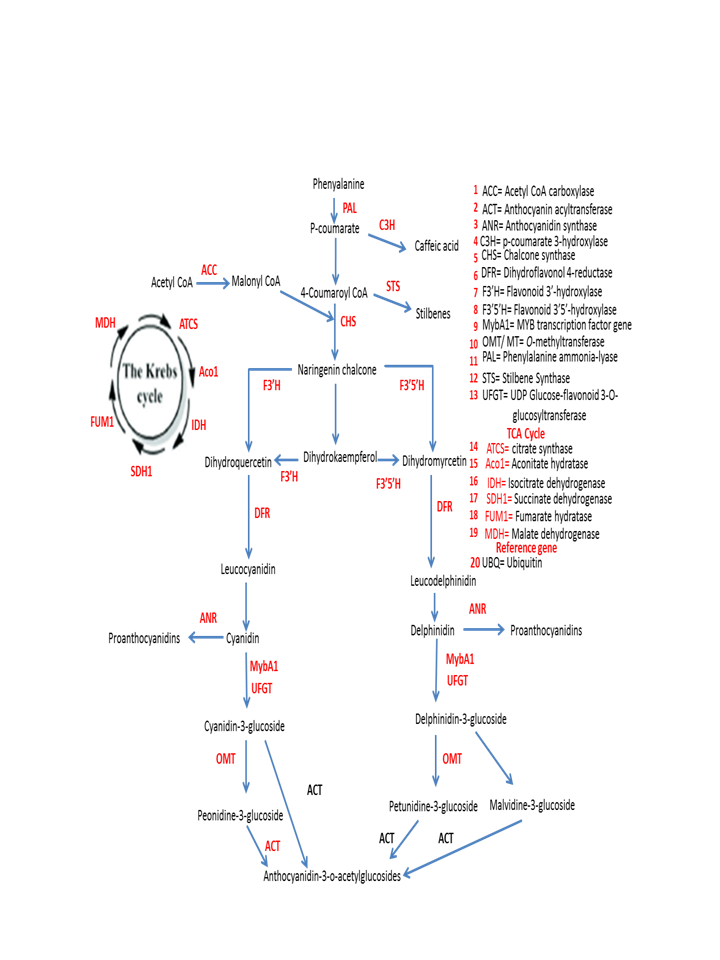

Supplement: FIGURE S1 — | Selected mediating enzymes and transcription factors in phenylpropanoid pathway and TCA cycle used in RT-qPCR based transcript analysis adapted from Zabala et al. (2006). ACC, Acetyl-CoA carboxylase 1-like; ANR, Anthocyanidin reductase; C3H, Coumarate 3-hydroxylase; CHS, Chalcone synthase; DFR, Dihydroflavonol 4-reductase; F3′H, flavonoid 3′ hydroxylase; F3′5′H, flavonoid 3′,5′ hydroxylase; MYB, Myb-related transcription factor; OMT, O-methyltransferase; PAL, Phenylalanine ammonia lyase; STS, Stilbene synthase; UFGT, UDP glucose-flavonoid 3-O-glucosyl transferase; ACo1, Aconitate hydratase 1-like; SDH1, Succinate dehydrogenase; ATCs, Citrate synthase; MDH, Malate dehydrogenase; FUM1, Fumarate hydratase; IDH, Isocitrate dehydrogenase; n = 3, mean values ± SE. [file Image_1.TIF]

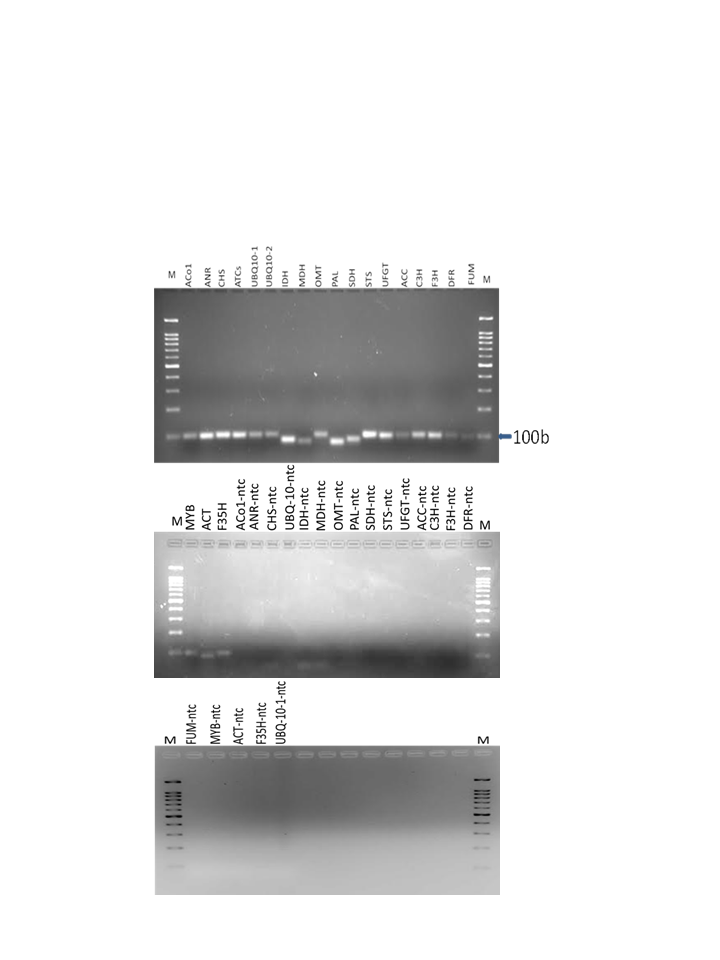

Supplement: FIGURE S2 — | Specificity of the designed primers for transcript analysis and their negative control, formaldehyde treated 1.2% agarose gel electrophoresis, each lane represents specific primers and ntc, lane 1 and 20 are size markers in all the three gels. First gel electrophoresis: ANR, CHS, ATCs, UBQ10-1, UBQ10-2, IDH, MDH, PAL, SDH, STS, UFGT, ACC, C3H, F3H, DFR, FUM from 2 till 19 sequentially, Second electrophoresis: MYB, ACT, F3′5H, ACo1-ntc, ANR-ntc, CHS-ntc, UBQ-10-ntc, IDH-ntc, MDH-ntc, OMT-ntc, PAL-ntc, SDH-ntc, STS-ntc, UFGT-ntc, ACC-ntc, C3H-ntc, F3H-ntc, DFR-ntc from 2 till 19 sequentially, third electrophoresis: FUM-ntc, MYB-ntc, ACT-ntc, F3′5′H-ntc, UBQ-10-1-ntc from 2 till 6 respectively. [file Image_2.TIF]
